# ENdb: a manually curated database of experimentally supported enhancers for human and mouse

**DOI:** 10.1093/nar/gkz973

**Published:** 2019-10-29

**Authors:** Xuefeng Bai, Shanshan Shi, Bo Ai, Yong Jiang, Yuejuan Liu, Xiaole Han, Mingcong Xu, Qi Pan, Fan Wang, Qiuyu Wang, Jian Zhang, Xuecang Li, Chenchen Feng, Yanyu Li, Yuezhu Wang, Yiwei Song, Ke Feng, Chunquan Li

**Affiliations:** School of Medical Informatics, Daqing Campus, Harbin Medical University. Daqing 163319, China

## Abstract

Enhancers are a class of *cis*-regulatory elements that can increase gene transcription by forming loops in intergenic regions, introns and exons. Enhancers, as well as their associated target genes, and transcription factors (TFs) that bind to them, are highly associated with human disease and biological processes. Although some enhancer databases have been published, most only focus on enhancers identified by high-throughput experimental techniques. Therefore, it is highly desirable to construct a comprehensive resource of manually curated enhancers and their related information based on low-throughput experimental evidences. Here, we established a comprehensive manually-curated enhancer database for human and mouse, which provides a resource for experimentally supported enhancers, and to annotate the detailed information of enhancers. The current release of ENdb documents 737 experimentally validated enhancers and their related information, including 384 target genes, 263 TFs, 110 diseases and 153 functions in human and mouse. Moreover, the enhancer-related information was supported by experimental evidences, such as RNAi, in vitro knockdown, western blotting, qRT-PCR, luciferase reporter assay, chromatin conformation capture (3C) and chromosome conformation capture-on-chip (4C) assays. ENdb provides a user-friendly interface to query, browse and visualize the detailed information of enhancers. The database is available at http://www.licpathway.net/ENdb.

## INTRODUCTION

Gene transcription is regulated by genomic elements, such as enhancers, promoters, silencers and insulators in a tissue-specific and spatiotemporal-specific manner (1,2). Among such elements, enhancers are a class of *cis*-regulatory elements that can facilitate the transcriptional activity of genes by forming loops in intergenic regions, introns and exons. A large number of enhancers, as well as their corresponding target genes and transcription factors (TFs), were found to be highly associated with human disease and biological processes (3). Active enhancers are always marked by chromatin marks (e.g. H3K4me1 and H3K27ac) and can be identified through high-throughput and low-throughput experimental methods (4–8). The most commonly used low-throughput methods include RNAi (9), in vitro knockdown (10), electrophoretic mobility shift assay (EMSA) (11), western blotting (12), qRT-PCR (13), luciferase reporter assays (14), chromatin conformation capture (3C) (15) and chromosome conformation capture-on-chip (4C) (16) assays. These assays have been used to directly and quantitatively assess enhancer activity, as well as that of their related regulatory elements. Low-throughput techniques are undoubtedly more accurate for the identification of enhancers compared with high-throughput techniques. Conversely, high-throughput experimental techniques provide approaches to investigate huge amounts of computationally-predicted enhancers and genome-wide features. To date, the human genome has been extensively cataloged with over one million candidate enhancers (17). Additionally, the number of publications on enhancers increases each year, which will advances in research will provide for collecting more reliable evidences of enhancer.

Some enhancer databases, as well as super-enhancer databases, have been published, including EnhancerAtlas (18), HACER (19), HEDD (20), EnhancerDB (21), DiseaseEnhancer (22) and EnDisease (23). These databases have provided valuable resources for enhancer investigation (24–33);however, most of them only focus on enhancers identified by high-throughput experimental techniques. Recognizing that potential false positives exist in high-throughput experiments, several low-throughput experimentally-supported enhancer databases were built (22,23,34). For example, the VISTA Enhancer Browser (34) is a central resource for experimentally-validated human and mouse fragments with gene enhancer activity assessed in transgenic mice. The VISTA Enhancer Browser group performed numerous transgenic mouse assays to determine the activity of putative enhancers. DiseaseEnhancer and ENdisease differ from VISTA Enhancer Browser in terms of their strategy of collecting enhancers. Those database manually curated low-throughput experimentally-supported enhancers by reviewing hundreds of publications. However, they are specific in their respective purposes (i.e. focus on diseases). Therefore, it is desirable to construct a comprehensive resource of manually curated enhancers and related information (e.g. target genes, TFs binding to enhancers, functions and diseases) derived from low-throughput experimental evidence.

Here, we established a comprehensive, manually curated enhancer database for human and mouse (ENdb, http://www.licpathway.net/ENdb), which aimed to provide a resource for experimentally-supported enhancers, and to annotate the details of such enhancers. Enhancers were manually examined from a review of 1,590 published studies, In general, if the genomic location of enhancers could be retrieved and enhancer activity could be experimentally validated, the enhancers were stored in ENdb. The current release of ENdb documents 737 experimentally validated enhancers and related information, including 384 target genes, 263 TFs, 110 diseases and 153 functions in human and mouse. ENdb provides a user-friendly interface to query, browse and visualize detailed information about enhancers. We hope that this elaborate database, which is supported by strong experimental evidence, can serve as an important catalyst for future research.

## DATA COLLECTION AND DATABASE CONTENT

To ensure high quality data collection, we referred to the steps involved in manual collection from other databases, such as DiseaseEnhancer (22), Lnc2Cancer (35) and EVLncRNAs (36). The abstracts of all publications with the keyword matching ‘enhancer,’ were initially retrieved from the PubMed database (37). We manually reviewed a total of 23 462 abstracts to obtain the pertinent enhancer-related publications for human and mouse. A total of 4641 publications associated with enhancers were retained for human and mouse. The following workflow was mainly applied for extracting enhancer information from the publications. First, we filtered 4601 publications based on two criteria: (i) the genomic location of enhancers could be obtained from the publications (e.g. from the figures, or from in-text descriptions); and (ii) the enhancers were supported by at least one form of experimental evidence. After filtering publications based on those criteria, 1590 publications were retained. Second, we reviewed the full text of the 1590 publications in detail and extracted the general information of enhancers and related information (e.g. target genes, TFs binding to enhancers, functions and diseases). Notably, general information of enhancers was extracted from the publications,including the PubMed ID of the publication, the enhancer name, chromosome and positional information, experimental techniques (e.g. luciferase reporter assay, EMSA, western blotting and 3C), experimental samples (cell line or tissue), the experimental class of enhancers (‘low-throughput’, ‘low- + high-throughput’), species (human or mouse) and reference genome (hg19 or mm10). Third, the enhancer-related information was also collected, including the TFs binding to enhancers, target genes, functions and diseases, as well as the type of experimental assay performed, such as luciferase reporter assay, qRT-PCR, RNAi, in vitro knockdown, western blotting, 3C and 4C. For example, the experimental evidence for TFs (e.g. ChIP-qPCR and EMSA) was used to confirm the binding of transcription factors to enhancers. The relationships (38) between enhancers and target genes were divided into those supported by ‘strong evidence’ and ‘less strong evidence’. If evidence was derived from 3C, 4C, 5C or CRISPR/Cas9, a label of ‘strong evidence’ was assigned. Otherwise, ‘less strong evidence’ was assigned. For example, RNAi used to knockdown transcripts of TFs that bind the enhancers to test expression of target genes. Thus, we considered RNAi as ‘less strong evidence’ for relationships between enhancers and target genes. Finally, we used a previously standardized classification scheme described (35) to map disease names to Disease Ontology (39) and MeSH (40) databases. The names of target genes and TFs associated with enhancers were replaced with official or recommended names. All enhancers and related information (e.g., target genes, TFs binding to enhancers, functions and diseases) were manually extracted from the selected publications by at least two researchers. Moreover, ENdb provided links to external resources, including PubMed ID in NCBI, HGNC, dbSNP and diseases in the Disease Ontology and MeSH databases.

During data collection, it was a challenge to extract information on the genomic locations of enhancers. However, we found that many publications provided important clues to determine such location information. Thus, by reviewing the figures, in-text descriptions provided in the publications, we could obtain accurate location information. For example, some enhancers and related genomic locations were only provided in the figures of publications. In such cases, we determined the location of enhancers by carefully reviewing the figures in the full text. For some cases where only single point within an enhancer, such as ‘enhancer locating at upstream xxx kb from the transcription start sites’, is provided in original publications, the ±1 kb region centered on this point was used as the genomic location of the enhancer (17). All genomic locations for human and mouse were unified in the corresponding hg19/mm10 genomic coordinates.

## DATABASE STATISTICS

**Figure 1.**
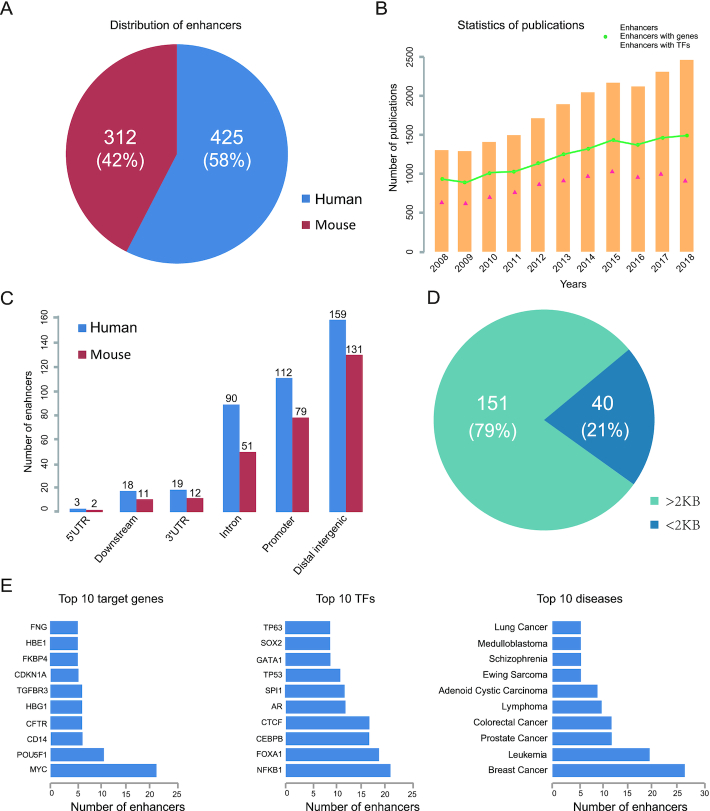
Statistics of enhancers in ENdb. (**A**) The distribution of enhancers for human and mouse. (**B**) The annual number of publications on enhancers and their related information each year. (**C**) The distribution of enhancers in genomic elements. (**D**) The distribution of enhancers in the promoter and distant regions. (**E**)The number of enhancers associated with the top 10 target genes (left), TFs (middle) and diseases (right).

The current version of ENdb contains 737 enhancers, 384 target genes, 263 TFs, 110 diseases, 153 functions and 34 SNPs for human and mouse. There are 425 enhancers for human and 312 for mouse (Figure 1A). We counted the number of publications each year from 2008 to 2018, that involved in enhancers, enhancers with information of target genes or those with TF binding data (Figure 1B). We found that the number of enhancer-related studies steadily increased over the past decade. Enhancers were mainly located in distal intergenic (39.7%), promoter (27.9%), intron (22.5%), downstream (4.4%), 3′UTR (4.8%), and merely containing 0.7% 5′UTR regions for human (Figure 1C). Mouse enhancers were mainly located in distal intergenic (45.8%), promoter (27.6%), intron (17.8%), 3′UTR (4.2%), downstream (3.9%) and 5′UTR (0.7%) regions (Figure 1C). Consistent with statistics from the DiseaseEnhancer database, most of enhancers were concentrated at the distal intergenic and near promoters. A possible explanation for enhancers being concentrated near promoters is that they are likely to be super-enhancers with large sizes or that they function as promoters in higher chromosomal organization. For enhancers located at promoter regions, we calculated the distance between the enhancer and the transcription start site of their target gene. The results revealed that the distances of only 40 (21%) enhancers was <2KB (Figure 1D). Thus, those data demonstrated that most enhancers located at promoter regions regulate distant genes, and do not enhance the closest gene.

The top 10 target genes ranked by the number of associated enhancers represented by 18% of the curated target genes associated with enhancers (Figure 1E). The proto-oncogene MYC was one of the top target genes ranked based on the number of associated enhancers, and was affected by 22 enhancers. For example, a NOTCH-bound MYC enhancer played essential roles in the T-Cell Acute Lymphoblastic Leukemia (41), while in colon cancer, MYC expression was concurrently regulated by enhancers and complexes (42). On average, 14 enhancers were associated with the top 10 TFs that were ranked based on the number of associated enhancers in the ENdb database (Figure 1E). A large number of TFs were also able to bind various enhancers and exert their functions (e.g. SPI1, CTCF, FOXA1, AR and TP53). The top 10 diseases ranked by the number of associated enhancers represented by 53.5% of the curated disease-associated enhancers. A total of 27 enhancers were involved in breast cancer, 20 in leukemia, and 12 in prostate cancer (Figure 1E).

## USER INTERFACE

**Figure 2.**
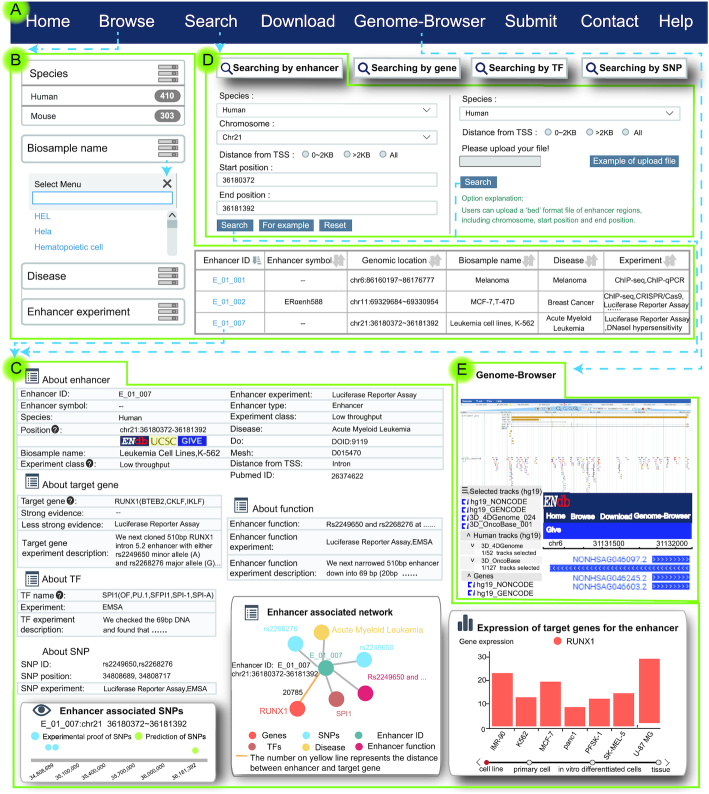
Database content and construction. (**A)** ENdb provides a user-friendly interface to browse, search, download, submit and visualize detailed information about enhancers. (**B**) The ‘Browse’ page allows users to quickly browse the information of enhancer and provides fuzzy search functions according to different conditions. (**C**) The ‘Detail’ page provides the general information of enhancer and the enhancer-associated information. The enhancer-associated information is graphically displayed in the page. (**D**) The ‘Search’ page allows users to search by a variety of keywords to return the records via four paths. Users also search the information of enhancers via the official symbols or aliases of genes. (**E**) Users view the proximity information of enhancers in the genome in the ‘Genome-Browse’ page.

ENdb provides a user-friendly interface to browse, search, download and visualize detailed information about enhancers (Figure 2A). The ‘Data-Browse’ page is organized as an interactive and alphanumerically sortable table that allows users to quickly browse through ‘Enhancer ID’, ‘Enhancer symbol’, ‘Genomic location’, ‘Biosample name’, ‘Enhancer experiment’ and ‘Disease’ (Figure 2B). The sub-menu of the ‘Browse’ page provides fuzzy search functions according to different conditions (Figure 2B). Simultaneously, users can browse enhancers by clicking Enhancer ID, and a list of matched entries is returned. The ‘Detail’ page contains the general information of enhancers, such as PubMed ID of publication, the enhancer symbol, chromosome and positional information, experimental techniques (e.g., luciferase reporter assay, EMSA, western blotting, 3C), the level of evidence for the relationships between enhancers and target genes (e.g., ‘strong evidence’ or ‘less strong evidence’), experimental samples (cell line and/or tissue), species (human or mouse) and reference gene (hg19 or mm10) (Figure 2B). The enhancer-associated network graphically displays the regulatory information of the enhancer, in which the relative positions of the enhancer and target genes are provided (Figure 2C). In the ‘Search’ page, ENdb allows users to search by a variety of keywords, facilitating searches by returning the records with the closest match. Users can search enhancers via four paths, including ‘Search by enhancer’, ‘Search by target gene’, ‘Search by TF’, ‘Search by SNP’ (Figure 2D). We also provide a function to search by gene name. Using this function, Users can input gene names, including gene official symbols or aliases to search the information associated with enhancers.

In order to help users intuitively view the proximity information of enhancers in the genome, ENdb provides two genome browsers (GIVE and JBrowse; Figure 2E) (43,44). Hundreds of tracks of histone modifications and chromatin interaction data can be browsed using the genome browser. GIVE enables automatic generation of interactive visualization webpages for user-selected chromosome conformation data. In addition, in the browse and search pages, users can link to UCSC (45), GIVE and Genome-Browse via the genomic position of enhancers. This allows users to visualize enhancer-related information, such as the relative positions of enhancers and target genes. ENdb allows users to download all the obtained data in the ‘Download’ page. In addition, ENdb also offers a submission page that enables researchers to submit novel experimentally-supported enhancers and their related information.

## SYSTEM DESIGN AND IMPLEMENTATION

The general workflow and features of ENdb was developed using MySQL (version 5.7.17) (https://www.mysql.com/). The ENdb was designed and the interactive interface was built using Bootstrap (version 3.3.7) and JQuery (version 2.1.1). The ECharts (http://echarts.baidu.com) and D3 (https://d3js.org) were used as a graphical visualization framework, and JBrowse (http://jbrowse.org) was used as the browser framework. We recommend using the database with a modern web browser that supports HTML5, such as Firefox, Google Chrome, Safari, Opera or IE 9.0+. ENdb is freely available to the research community using the web link provided (http://www.licpathway.net/ENdb). Users are not required to register or login to access features in the database.

## DISCUSSION

Enhancers are critical elements for regulating the transcription, and influence the expression of genes, as well as developmental and disease-related processes. In recent years, several projects have been developed, including ENCODE (17), FANTOM5 (25), Roadmap (26) and Blueprint/IHEC (46). Many candidate enhancers have been identified and enhancer databases have been established to provide a resource for collecting the detailed information for such enhancers. However, most of those databases focus on enhancers identified by high-throughput experimental techniques, while low-throughput techniques (e.g. RNAi, EMSA, western blotting, qRT-PCR, luciferase reporter assay, 3C and 4C) are more accurate in identifying enhancers and in exploring their upstream/downstream regulatory mechanisms. Therefore, we developed ENdb, a comprehensive database of all enhancers that were validated by low-throughput experiments in human and mouse.

We compared ENdb to the other three experimentally validated enhancer-related databases (VISTA Enhancer Browser, DiseaseEnhancer and ENdisease), and the results showed that 629 (85.3%) enhancers were uniquely collected in our database. The VISTA Enhancer Browser is an experimentally validated enhancer database, in which gene enhancer activity is accessed in transgenic mice (34). The VISTA Enhancer Browser group performed many transgenic mouse assays to determine the activity of putative enhancers based on candidate regions from seven publications. However, DiseaseEnhancer and ENdisease focus on manually curated enhancers associated with diseases from a large number of publications (22,23). Compared to DiseaseEnhancer and ENdisease, ENdb adopted a strict strategy to collect 737 (human, mouse) enhancers validated by low-throughput experiments from >1000 publications. Notably, the genomic location of enhancers must be retrievable from publications to be included in ENdb. Enhancer activity must also be directly validated by low-throughput experiments for inclusion in the ENdb. Many enhancers in DiseaseEnhancer were indirectly collected through mapping single nucleotide variants (SNVs) to enhancers identified by high-throughput experiments (22). SNV functions were validated by biological experiments. Additionally, ENdisease contains enhancers validated by both low and high-throughput experiments. However, the number of enhancers validated by low-throughput experiments is 434, which is less than that contained in ENdb. More importantly, compared to other databases, ENdb focuses on collecting experimentally-validated enhancers and associated information, including target genes, TFs, diseases and functions. Thus, ENdb has considerable potential to complement other enhancer-related databases.

Enhancers and super-enhancers, can drive high expression of genes involved in cell identity and disease. For example, super-enhancers frequently regulate lineage specific genes that control cell state and differentiation in somatic cells (47). In cancer cells, enhancers drive the expression of critical oncogenes, such as MYC (48), RUNX1 (49), MYOD (50) and BCL6 (51). Interestingly, negative regulators of enhancer-associated genes including the TFs CEBPA, IRF8, IRF1 and ETV6, can suppress cancer cell growths (52). Tsukiji *et al.* found that the expression of Shh was achieved through a novel regulatory element comprised of TFs and multiple enhancers in mouse embryonic endodermal tissues (53). Importantly, emerging evidence suggests that disease-associated SNPs are preferentially enriched in enhancers and can make essential contributions to disease progression (54). The causal SNP, rs58692659, in enhancer regions disrupts DNA-binding and enhancer activity (55). Together, these studies demonstrate the importance of enhancers and associated annotation information in human disease and biological processes. Our effort to establish ENdb was prompted by the great need of researchers to access a comprehensive dataset of experimentally validated enhancers and associated information, including genomic location, target genes, TFs, functions and diseases. Researchers in need of such a comprehensive database include cellular/molecular biologists, geneticists and data scientists. In the future, we will continue to manually curate newly validated enhancer associations and update the database. We will also consider continuously adding experimentally-supported enhancers from different species. We believe that ENdb can serve as a reliable and valuable resource of enhancers information to understand human diseases and biological processes.
